# Osteoarthritis Progression after ACL Reconstruction Was Significantly Higher Than That of the Healthy Contralateral Knees: Long-Term Follow Up Study of Mean 16.4 Years

**DOI:** 10.3390/jcm11030775

**Published:** 2022-01-31

**Authors:** Ali Zadehmohammad, Johannes Grillari, Vlado Stevanovic, Georg Brandl, Lukas Ernstbrunner, Thomas Hoffelner

**Affiliations:** 1 Department of Orthopedics and Traumatology, Paracelsus Medical University Hospital, Strubergasse 21, 5020 Salzburg, Austria; thomas.hoffelner@kh-herzjesu.at; 2 Ludwig Boltzmann Institute for Experimental and Clinical Traumatology, Donaueschingerstraße 13, 1200 Vienna, Austria; johannes.grillari@trauma.lbg.ac.at; 3 Department of Radiology, Paracelsus Medical University Hospital, Muellner Hauptstraße 48, 5020 Salzburg, Austria; v.stevanovic@salk.at; 4 Department of Orthopedic Surgery, St. Vincent Shoulder & Sports Clinic, Baumgasse 20A, 1030 Vienna, Austria; georg.brandl@kh-herzjesu.at; 5 Department of Orthopaedic Surgery, Royal Melbourne Hospital, Parkville, VIC 3050, Australia; lukas.ernstbrunner@icloud.com; 6 Melbourne Orthopaedic Group, Windsor, VIC 3181, Australia; 7 Department of Biomedical Engineering, University of Melbourne, Parkville, VIC 3010, Australia

**Keywords:** knee, osteoarthritis, anterior cruciate ligament, reconstruction, athletes, long term follow-up

## Abstract

Background: This study aimed to assess long-term progression of osteoarthritis (OA) after isolated anterior cruciate ligament (ACL) reconstruction in athletes compared to the healthy contralateral side. Methods: The study included 15 patients and 30 knees with a mean age of 40 years (range, 35–46) years, none of whom had had revision surgery or an injury to the contralateral side. The mean follow-up period was 16.4 years (range, 13–22). Clinical and radiographic assessment included the Tegner activity scale (TAS), International Knee Documentation Committee (IKDC) score, Knee injury and Osteoarthritis Outcome Score (KOOS), and Kellgren and Lawrence (KL) grade. The long-term results of the injured knees were compared with the status of the healthy contralateral side and compared with previously published mid-term results of the same cohort of patients. Results: Patients generally remained clinically asymptomatic or mildly symptomatic at final follow-up, which is reflected by a KOOS pain score of 33 points (maximum 36 points) and an IKDC total subjective score of 87% (maximum 100%). There was a significant difference between mid-term and final follow-up in terms of the function score of the IKDC subjective questionnaire (*p* = 0.031), compartment findings and donor site morbidity of the IKDC functional examination (both *p* = 0.034), and the total KOOS score (*p* = 0.047). The KL score indicated significant progression of OA from mid-term to final follow-up in the injured knees (*p* = 0.004) and healthy contralateral knees (*p* = 0.014). Mean OA grades of the injured knees were significantly higher compared with the healthy contralateral side (*p* = 0.006) at final follow-up, and two patients showed moderate to severe signs of OA in the injured knee. Conclusions: Although most patients remained clinically asymptomatic or mildly symptomatic, long-term progression of OA after isolated ACL reconstruction in athletes was significantly higher compared with the healthy contralateral knee.

## 1. Introduction

Non-operative management of torn anterior cruciate ligament (ACL) can be associated with the development of osteoarthritis (OA) [1], especially when there are concomitant meniscal or multiligament injuries [2], and they are more frequently observed as long-term sequelae in athletes [3,4]. There is an ongoing debate about whether the risk of developing OA in conservatively treated ACL tears is higher than after ACL reconstruction due to the resulting rotational instability, which can lead to higher meniscal and cartilage shear forces [5,6,7].

The current recommendation for isolated ACL injuries favors surgical management in athletes [8]. Whether ACL reconstruction for an isolated injury can alter the development and progression of OA in athletes is debatable [9], as certain long-term studies reported a delay in the degenerative process [10,11], whereas other long-term studies showed a progression of OA [12,13]. Our initial results of isolated ACL reconstruction in athletes after a mean of 7.8 years showed no significant increase in OA in the injured knee compared to the uninjured knee [3]. Whether these results remain stable over a longer-term period is uncertain. Hence, the purpose of this study was to assess the long-term progression of OA in the same cohort of athletes following ACL reconstruction for an isolated injury and to compare it to our mid-term results, as well as to the uninjured contralateral knee. We hypothesized that OA does not significantly increase over a 16-year observational period and that the rate of OA in injured knees is not significantly different compared with uninjured knees.

## 2. Materials and Methods

Between 1995 and 2005, 28 athletes underwent ACL reconstruction for an isolated injury performed by two experienced orthopedic surgeons. Grafts used were either a bone−patellar tendon−bone (BPTB) graft or a hamstring tendon (HT) graft. Only patients without revision surgery, additional injuries such as meniscal tears and an uninjured contralateral knee were included in this study. At final follow-up, of the initial 28 patients [3], seven (25%) had revision surgery, and four (14%) were diagnosed with meniscal injuries in the uninjured knee. Two patients (7%) were lost to follow-up.

Therefore, the study cohort consisted of 15 athletes (nine men (60%) and six women) with a mean age of 40 (range = 35–46) years. The mean follow-up period was 16.4 (range = 13–22) years. The clinical and radiographic results were compared to our previous results at a mean follow-up of 7.8 (range = 4–13) years [3].

Ethical approval was granted by the Ethics Committee of Salzburg (date: 24 February 2017/no. 415-E/2124/4-2017), and all included patients gave their consent for this study.

### 2.1. Surgical Technique

All 15 patients had undergone arthroscopically assisted ACL reconstruction, six with a BPTB graft and nine with an HT graft (double-looped single-bundle) (Table 1). Under general anesthesia, the Lachman, anterior drawer, and pivot-shift tests were used to confirm the magnetic resonance imaging (MRI)-based diagnosis of an ACL tear. ACL reconstruction was performed according to the surgical technique described by Hoffelner et al. [3]. The tibial tunnel was positioned in the medium range of the intercondylar area between the medial and lateral tubercle by use of a tibial tunnel guiding device (Arthrex, Naples, FL, USA). The femoral tunnel was drilled through the tibial tunnel with the knee flexed at 90° by use of a transtibial femoral guiding device (Arthrex, Naples, FL, USA). The femoral screw was placed on the lateral femoral condyle at the 10- to 11-o’clock position in the right knee and the 1- to 2-o’clock position in the left knee.

For the hamstrings, femoral fixation was performed with the EndoButton device (Smith & Nephew, Andover, MA, USA). Tibial fixation was carried out with a bioabsorbable interference screw (BIORCI Interference Screw; Smith & Nephew, Andover, MA, USA).

For the patellar tendon, a standard BPTB autograft was harvested from the ipsilateral side of the knee. Femoral fixation was performed with the RigidFix femoral fixation device (DePuy Mitek, Raynham, MA, USA). For tibial fixation, an interference screw was used [3].

### 2.2. Clinical and Radiographic Assessment

One examiner (AZ) performed the clinical assessment, which included a functional investigation according to International Knee Documentation Committee (IKDC) guidelines [14], an IKDC questionnaire for assessing subjective outcomes, the Knee injury and Osteoarthritis Outcome Score (KOOS) [15], and sports activity level using the Tegner activity scale (TAS) [16]. As part of the IKDC guided functional examination, the single legged hop test [17] and the knee walking test for evaluation of donor site morbidity [18] were conducted. According to the IKDC, the Lachman and pivot shift test were performed and included in the group grading. The percentage of attained scores in the IKDC questionnaire for assessing subjective outcome was declared as total IKDC % [14].

Joint space narrowing in the injured and uninjured knees was radiographically evaluated using weight-bearing anteroposterior Rosenberg comparative views [19]. The Kellgren and Lawrence (KL) scoring system was used to assess OA [20]. This grading system has a maximum attainable score of 4 points: 0 = no sign of OA; 1 = slight narrowing of joint space and possible osteophytic lipping; 2 = definite osteophytes and joint space narrowing; 3 = multiple osteophytes, definite joint space narrowing, slight sclerosis and possible deformities of bone contour; and 4 = large osteophytes, severe joint space narrowing and sclerosis as well as definite deformity of bone contour [20]. Following, 3T MR (Philips Achieva; Philips Medical Systems, Andover, MA, USA) images of both knees were obtained. The 3D water-selective cartilage (WATS-c) scan sequence (slice thickness = 1 mm) was used to for calculation of the International Cartilage Repair Society (ICRS) scoring system [21] to enable comparison with our previous results [3]. Imaging analyses of bone cysts, osteophytes and lesions to the posterior horn of the medial meniscus were also conducted.

Two of the authors (A.Z. and V.S.), who were blinded to clinical and radiographic results, analyzed the images and reached a consensus.

### 2.3. Statistics

Normal distribution was tested using the Shapiro–Wilk test. A paired comparison using the t test (normal data) and the Wilcoxon signed rank test (non-normal data) was conducted to find significant differences between our previous (mid-term) and current long-term results of the injured knee, as well as between the injured and uninjured knee at final follow-up. The α level was set to 0.05, and all *p* values were two-tailed.

## 3. Results

### 3.1. Clinical Assessment

Patients remained clinically asymptomatic or mildly symptomatic at final follow-up, which is reflected by a KOOS for pain score of 33 points (maximum 36 points) and an IKDC total subjective score of 87% (maximum 100%). There was a significant difference between mid-term and final follow-up in terms of the function score of the IKDC subjective questionnaire (*p* = 0.031), compartment findings and donor site morbidity of the IKDC functional examination (both *p* = 0.034), and the total KOOS score (*p* = 0.047). All other clinical outcome measures showed no significant differences comparing the mid-term results with the results at final follow-up (Table 2).

When compared to the healthy contralateral side, the IKDC functional examination of the injured knee showed that 13 patients (86.7%) had a normal range of motion, and two patients (13.3%) showed nearly normal motion. In the one-leg hop test, 12 patients (80.0%) jumped on the injured leg to a height that was ≥90% of the height reached on the contralateral leg, while the other three patients (20.0%) were able to hop to a height 76–89% of the height reached on the contralateral leg. When performing the knee walking test for donor side morbidity, six patients (40%) reported a normal sensation, five (33.3%) reported being uncomfortable, and four (26.7%) found it difficult. Patients who received a BPTB graft seemed to have more symptoms while performing activities of daily living based on the KOOS subscore versus patients who received an HT graft (*p* = 0.027), but there were no significant differences in other clinical outcome scores between the two groups.

### 3.2. Radiographic Assessment

At final follow-up, the KL score indicated mild OA (grade 2) in three injured knees (20.0%) and in one uninjured knee (6.7%). Moderate OA (grade 3) was seen in the injured knee of one patient (6.7%), while severe OA (grade 4) was also detected in one injured knee (6.7%). No radiographic signs of OA were observed in the remaining 10 injured knees and in the remaining 14 uninjured knees. Overall, the mean OA grades of the injured knees were significantly higher compared with the uninjured knees (*p* = 0.006). Compared with the KL scores at mid-term follow-up, both the injured and uninjured knees showed significant progression of OA at final follow-up (injured, *p* = 0.004; uninjured, *p* = 0.014). This significant progression of OA over time in the injured knees was also confirmed by the MR-based assessment using the guidelines of the ICRS (*p* = 0.013), but it showed no significant progression of OA in the uninjured knee (Table 3).

When comparing OA of the injured versus the uninjured knees at final follow-up, the KL score revealed significantly higher OA grades in the injured knees (1.3 ± 1.1 vs. 0.6 ± 0.6; *p* = 0.006) and a tendency toward higher OA grades in the injured knees based on the ICRS guidelines (1.9 ± 1.5 vs. 1.3 ± 1.4; *p* = 0.055).

## 4. Discussion

The main finding of this study is that long-term progression of OA after isolated ACL reconstruction in athletes was significantly higher compared with the healthy contralateral knee, which refuted our second hypothesis that the rate of OA in injured knees is not significantly different compared with uninjured knees in athletes undergoing ACL reconstruction for an isolated injury. In addition, our second hypothesis had to be refuted, as there was significant long-term progression of OA after isolated ACL reconstruction, although a similar progression was also observed in the uninjured knees. Besides this clear radiographic progression of OA and more pronounced OA in the injured knee at long-term follow-up, most patients remained clinically asymptomatic or mildly symptomatic.

Current long-term studies report controversial clinical and radiographic outcomes after ACL reconstruction. Some studies reported a delay in OA progression after surgery [1,11,22]. Kvist et al. [1] followed 153 patients, including athletes and non-athletes, who had an ACL tear and received either conservative or surgical treatment. Patients allocated to early surgery had lower rates of OA after 32 to 37 years compared to patients treated conservatively. However, patients had similar clinical outcomes in both treatment groups. This is in contrast to other studies that suggested an increase in the degenerative process after ACL reconstruction over time [23] or compared to conservative treatment [24,25]. Kessler et al. [24] evaluated 109 patients treated conservatively or surgically, and patients who received ACL reconstruction showed more pronounced radiographic signs of OA after 11 years as compared to the ACL-deficient group. In addition, the clinical outcome according to the IKDC score was better in the conservatively managed group. There are clear differences in our results compared to these studies, such as they did not focus on isolated injuries to the ACL, nor were only athletes included. We also did not compare our results to a non-operatively treated group, but similar to some of the previous studies, besides the observed significant progression of OA in the surgically treated knee versus the healthy contralateral side, subjective clinical outcome was good at final follow-up. It remains unclear whether the observed radiographic deterioration over time is prodromal for a transition from mildly symptomatic to clinically relevant OA in the ACL reconstructed knees regarding the future.

Similar to our study, Holm et al. [26] examined the differences between the KL scores of the ACL reconstructed and uninjured knee and obtained results comparable to ours: a higher incidence of OA in the injured knee. Although cartilage degeneration is a physiological process of aging, multiple factors have been shown to be associated with acceleration of this process. Such factors include age, sex, obesity, genetics, occupation, and trauma [27]. It is also known that athletes involved in intense and strenuous activities are more likely to be affected by OA, particularly in the knee [28,29,30,31]. ACL reconstruction has not been shown to prevent OA directly, but it is considered to play a major role in restoring rotational stability, thus preventing further injury to the knee joint, which would promote degenerative processes [32]. After a 10-year period, the risk of developing OA may be reduced by initial ACL surgery [22]. However, the surgical procedure itself may also play a role in enhancing OA in the knee joint even in minimally invasive arthroscopy.

Selecting young patients for this study was crucial for evaluating OA outcome parameters because patients under 35 years of age are at a higher risk of developing OA after ACL injury [33]. The population of our study may show a higher rate of radiographic signs of OA in both knees as they get older because of the natural ongoing degenerative processes in the knee joint. The natural course of OA in the knee is significantly influenced by repetitive, high-impact activity. Hence, an increase in the degenerative process in both knees of this patient group according to the KL scores appears logical.

Considering that clinical symptoms such as pain are important in diagnosing OA in the knee, our patient group had a satisfactory clinical outcome after a mean of 16.4 years after isolated ACL reconstruction. Tesarz et al. [28] performed a systematic review of 15 articles and concluded that athletes had a significantly higher level of pain tolerance than did normally active controls. This conclusion might be important when interpreting results and correlating them with degenerative changes. Pinczewski et al. [34] concluded that the radiographic signs of OA in their study population were subclinical after ten years. Their patients were highly active at the time of injury. The subjective results of Pinczewski et al. appeared comparable to those of our patient group.

Although all available patients were included in this study, there are inevitable limitations associated with this retrospective design, such as limited power. Notwithstanding, a power calculation was omitted in this study, as post hoc power analysis is an inappropriate tool for power estimates [35,36]. In addition, no baseline imaging or clinical assessment was performed before the injury and surgery, although cartilage abnormalities in this healthy and previously pain-free population would have been unlikely.

## 5. Conclusions

Although most patients remained clinically asymptomatic or mildly symptomatic, long-term progression of OA after isolated ACL reconstruction in athletes was significantly higher compared with the healthy contralateral knee.

## Figures and Tables

**Table 1:** Demographic data of athletes separated by graft type.

Patient Characteristics	BPTB	HT
Number of patients	6	9
Age in years (mean ± SD)	40 ± 3.37	41 ± 4
Number of soccer players	6	4
Number of skiers	0	5
Years of follow-up (mean ± SD)	18.3 ± 2	15.1 ± 2.7
Body mass index (mean ± SD)	25.6 ± 3.7	25.9 ± 3.4

BPTB, none–patellar tendon–bone; HT, hamstring tendon.

**Table 2:** Clinical and radiographic outcome at mid-term and final follow-up.

Variable ^†^	Mid-Term FU	Final FU	*p* (Mid-Term vs. Final FU)
TAS, points	6.1 ± 1.8	5.3 ± 1.2	0.183
IKDC function, points			
Effusion	0	0.3 ± 0.7	0.157
Motion deficit	0.1 ± 0.3	0.1 ± 0.4	0.564
Compartment findings	0.1 ± 0.3	0.5 ± 0.7	0.034 ^1^
Harvest site pathology	0.2 ± 0.4	0.6 ± 0.5	0.034 ^1^
One leg hop test	0.3 ± 0.5	0.2 ± 0.4	0.317
IKDC subjective, points			
Symptoms	30.7 ± 5.4	30.9 ± 5.8	0.487
Sports and recreation	36.7 ± 3.7	36.7 ± 3.2	1.000
Function	9.2 ± 1.4	8.4 ± 2.0	0.031 ^1^
IKDC total, percentage	88.1 ± 11.3	87.3 ± 11.4	0.733
KOOS, points			
Symptoms	23.7 ± 3.8	21.9 ± 5	0.145
Pain	34 ± 3.4	32.9 ± 4.2	0.322
ADL	67 ± 2.1	66 ± 2.7	0.169
Sport/Rec	17.5 ± 4.5	16.1 ± 4.4	0.108
QoL	13.4 ± 3	12.7 ± 2.7	0.227
KOOS total, percentage	92.6 ± 8.6	89.1± 9.5	0.047 ^1^

FU, follow-up; TAS, Tegner Activity Scale; IKDC, International Knee Documentation Committee; KOOS, Knee injury and Osteoarthritis Outcome Score; ADL, activities of daily living; Sport/Rec, sports and recreation; QoL, quality of life. ^†^ Data are presented as mean ± standard deviation. ^1^ Significant result (*p* < 0.05).

**Table 3:** Radiographic outcome at mid-term and final follow-up.

Variable ^†^	Mid-Term FU	Final FU	*p* (Mid-Term vs. Final FU)
KL score injured, points	0.4 ± 0.6	1.3 ± 1.1	0.004 ^1^
KL score uninjured, points	0.2 ± 0.6	0.6 ± 0.6	0.014 ^1^
ICRS injured, points	1.1 ± 1.7	1.9 ± 1.5	0.013 ^1^
ICRS uninjured, points	1.1 ± 1.4	1.3 ± 1.4	0.164

FU, follow-up; KL, Kellgren and Lawrence; ICRS, International Cartilage Repair Society. ^†^ Data are presented as mean ± standard deviation. ^1^ Significant result (*p* < 0.05).
